# Racial Disparities in Clinical Trial Enrollment Among Patients Diagnosed With Prostate Cancer: A Population-Based Cohort of Oncology Practices

**DOI:** 10.1155/aiu/8871425

**Published:** 2024-12-19

**Authors:** Brett M. Wiesen, Thomas W. Flaig, Boris Gershman, Badrinath Konety, Adam Warren, Elizabeth Molina Kuna, Tyler Robin, Elizabeth R. Kessler, Corbin J. Eule, Benjamin N. Breyer, Justin Achua, Simon P. Kim

**Affiliations:** ^1^Department of Surgery, Division of Urology, University of Colorado, Aurora, Colorado, USA; ^2^Division of Medical Oncology, University of Colorado, Aurora, Coloarado, USA; ^3^Division of Urologic Surgery, Harvard Medical School, Beth Israel Deaconess Medical Center, Boston, Massachusetts, USA; ^4^Department of Urology, Allina Health, Minneapolis, Minnesota, USA; ^5^Population Health Shared Resource (PHSR) Department, University of Colorado Cancer Center, Aurora, Colorado, USA; ^6^Department of Radiation Oncology, University of Colorado, Aurora, Colorado, USA; ^7^Department of Urology, University of California San Francisco, San Francisco, California, USA

**Keywords:** clinical trial, outcomes, prostate cancer, racial disparities

## Abstract

**Background:** Although clinical trials should be accessible to all patients, persistent racial and ethnic disparities in clinical trial enrollment exist. Herein, we examine racial disparities in clinical trial enrollment among prostate cancer patients from a large population-based cohort of oncology practices in the United States.

**Methods:** Using CancerLinQ Discovery, we identified men with regional (N1+) and/or metastatic (M1) prostate cancer diagnosed from 2011 to 2023. Enrollment into a clinical trial for prostate cancer was the primary outcome. Multivariable logistic regression and Cox proportional hazard regression were used for analysis.

**Results:** Within our dataset, we identified 17,028 patients with advanced prostate cancer, of which only 2.6% of patients were enrolled in a clinical trial (*n* = 450). There was variance in the proportion of patients accrued over time with a low of 0.30% in 2011 to a high of 3.94% in 2018 and decreasing to 2.37% in 2023. On multivariable analysis, older age was associated with lower odds of clinical trial enrollment (*p* < 0.001). Compared to White patients, Hispanics/Latino (OR: 0.35; CI: 0.161–0.744, *p*=0.04) and patients with self-identified other race or ethnicity (OR: 0.23; CI: 0.295–0.931, *p* < 0.01) had lower odds of clinical trial enrollment on multivariable analysis. Black men with prostate cancer did not have a statistically significant difference compared to White men for clinical trial enrollment. (OR: 1.033; CI: 0.771–1.384, *p*=0.828).

**Conclusion:** While clinical trial enrollment remains low for men with advanced prostate cancer in this contemporary population-based cohort, rates of participation for Hispanic/Latino men, but not Black men, are significantly lower. Increased attention is needed to better understand the reasons behind these racial disparities and to develop effective interventions to promote access.

## 1. Introduction

Prostate cancer remains one of the most commonly diagnosed malignancies in males with approximately 268,490 incident cases and 34,500 cancer-related deaths each year in the United States [1]. Recent changes in the clinical practice guidelines based on the unclear results from two clinical trials on prostate cancer screening have been associated with a rising incidence of men presenting with metastatic prostate cancer and a higher proportion of men demonstrating high-risk, adverse pathologic features following surgery [2]. With the rising incidence of aggressive forms of prostate cancer, there is a need for increased awareness and advancement in treatment modalities.

Clinical trials represent an essential component to effectively evaluate diagnostic tests or therapeutic interventions in comparative effectiveness for cancer patients. Clinical trials play a crucial role in advancing medical knowledge and improving patient care. The benefits of clinical trials include the development of new treatments, drugs, and medical devices, which can lead to improved survival rates and quality of life for patients. Participants in clinical trials often gain access to cutting-edge treatments before they are widely available. In addition, the rigorous testing and monitoring involved in clinical trials ensure that new treatments are both safe and effective. However, there are potential harms associated with clinical trials, such as adverse side effects from experimental treatments, the possibility of receiving a placebo instead of an active treatment, and the psychological impact of participating in a trial. Ethical concerns also arise regarding informed consent and the potential exploitation of vulnerable populations. Balancing these benefits and risks is essential to conducting ethical and effective clinical research. Consequently, it is essential for accrual to clinical trials to include a diverse patient population to ensure that the results are generalizable to the population at large. Yet, prior studies have consistently demonstrated a significant underrepresentation of African American and Hispanic/Latino patients for clinical trials and prostate cancer trials in particular [3–5]. In response, the National Institutes of Health (NIH) has introduced multiple initiatives to encourage the participation of minority patients and expanded the diversity of clinical trial enrollment with recent federal legislation requiring a diversity plan in 2022 [6, 7].

Achieving greater diversity and inclusion in clinical trial participation requires a variety of policies and incentives for sponsors and pharmaceutical companies including accurate estimates of clinical trial accrual to best inform health policy. There is also a need for active recruitment and awareness by physicians to ensure better generalizable studies. Several key studies have demonstrated decreased enrollment compared to White men for African American, Latino, and Asian–American men with prostate cancer [4, 8, 9]. To characterize the lack of racial diversity within these trials, studies have been required to either report the racial composition of different clinical trials or rely on geographic regions, such as the surveillance, epidemiology, and end result (SEER), to define the at-risk patient population. Relying on SEER data or specific geographic regions can lead to inaccurate representation of racial diversity in clinical trials due to regional demographic differences and limitations in data granularity. These methods may obscure the true extent of diversity and fail to capture barriers faced by racial minorities, leading to an incomplete understanding of participation disparities. To address these issues, diverse data sources and targeted recruitment efforts are essential [10].

Addressing the gaps in knowledge is needed to develop effective interventions to improve equitable access and diverse accrual into clinical trials for advanced prostate cancer. Since large subsets of clinical trial enrollees appear to be recruited from nonacademic, community-based practices, it would be ideal to estimate enrollment distribution by race using the data drawn from both the academic and community sources. In this context, we sought to elucidate the contemporary trends in clinical trial enrollment by race for men with advanced prostate cancer and identify factors associated with enrollment from a large population-based cohort from oncology practices across the United States.

## 2. Methods

### 2.1. Patient Population

This study utilized the American Society of Clinical Oncology (ASCO) CancerLinQ Discovery® Database (CLQ-DB) to create a population-based, retrospective cohort of men diagnosed with advanced or metastatic prostate cancer [11]. CLQ-DB collects data from electronic health records from participating oncology practices. The data are standardized and undergo regular updates through robust quality control, encryption, and governance frameworks to advance cancer treatment and patient care. Participating oncology practices, researchers and providers, regulatory agencies, and policymakers may obtain access to CLQ-DB. The CLQ-DB contains longitudinal information regarding demographics, diagnosis, stage, treatments, laboratory values, and provider notes across over 80 community and academic oncology practices in the U.S. To create our analytic cohort, we identified all men aged 40–90 years who presented with or progressed to advanced prostate cancer between 2011 and 2023. Advanced prostate cancer was defined as regional lymph node involvement and/or the presence of distant metastasis. Patients may have received initial primary therapy with radical prostatectomy or radiation therapy and prior androgen deprivation therapy (ADT). 5845 patients underwent surgical therapy and 11,183 underwent radiation therapy with or without ADT. Our study was also considered as an exempt research by the Colorado Multiple Institution Review Board.

### 2.2. Covariates and Outcomes

We assessed patient and clinical covariates for evaluation in association with the primary outcome of our study, and enrollment into a clinical trial focused on prostate cancer during the study interval. Clinical covariates evaluated were patient age at diagnosis, race, geographic region, clinical TNM stage, Gleason score, and PSA at the time of diagnosis and year of diagnosis. For the race subcohort, White is defined as non-Hispanic White, Black is defined as non-Hispanic Black, and the Hispanic ethnicity includes any racial category that selects Hispanic or Latino as their ethnicity. CLQ-DB provides information about the date of clinical trial enrollment, which represented the primary outcome of our study. Within the time of the study, there was a range of 180–450 active clinical trials that specifically focused on prostate cancer. There was an increasing trend of trials available to patients throughout the interval.

### 2.3. Statistical Analysis

Bivariate associations between patient's clinical and pathologic characteristics with the outcome of clinical trial accrual were tested by using Pearson's chi-square test. Multivariable logistic regression analyses were performed to identify patient clinicopathologic covariates associated with clinical trial enrollment. SAS Version 9.4 (SAS Institute Inc., Cary, North Carolina) was used to perform all statistical analyses with a two-sided *p* value of < 0.05 that was used to determine statistical significance.

## 3. Results

**Figure 1:**
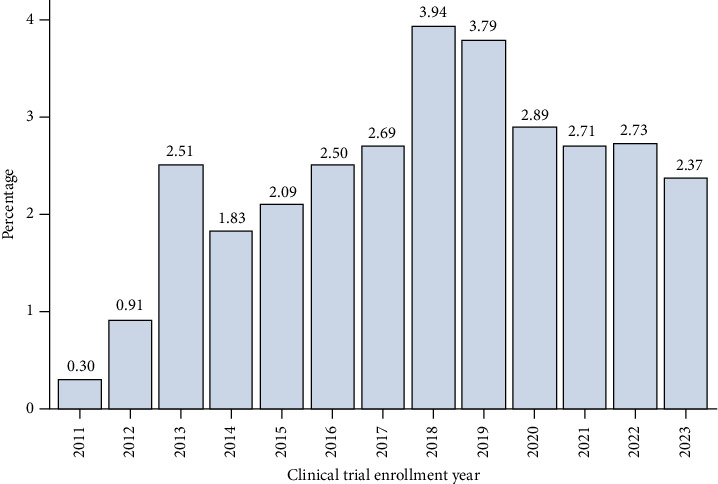
Proportion of patients enrolled into clinical trial over time.

**Figure 2:**
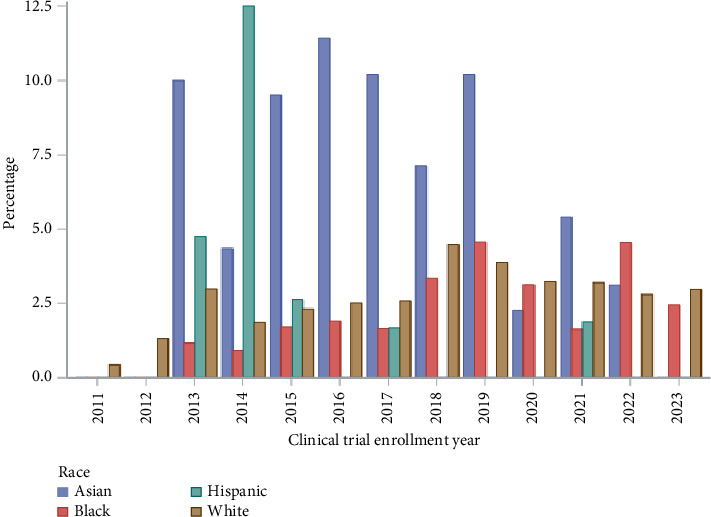
Proportion of patients enrolled into clinical trial by race over time.

During the study interval, we identified 17,028 patients who presented with advanced or metastatic prostate cancer. The median follow-up was 77 months (95% CI: 75–80). The mean follow-up was 83.5 months (SD: 0.518 months). Overall, we found only 2.6% of patients were enrolled into a clinical trial focused on prostate cancer (*n* = 450). There also was an increase in the trend of available clinical trials during the study interval.  Table 1 presents the clinical and demographic characteristics of the analytic cohort. On bivariate analysis, we observed differences in clinical trial enrollment across age group, race, and geographic region. The proportion of patients accrued varied over time but remained low overall from 0.30% in 2011 to a high of 3.94% in 2018 and decreasing to 2.37% in 2023 (Figure 1; *p* < 0.001 for trend). Our results also demonstrate that clinical trial enrollment varied over time by race as well (Figure 2; *p* < 0.001 for trend). More specifically, racial disparities for lower enrollment into clinical trials existed over time for Hispanic/Latino men and men who identified as a race or ethnicity designated as other but not Black men.

On multivariable analysis, several patient characteristics were associated with clinical trial participation (Table 2). For example, those aged ≥ 80 years old were associated with lower adjusted odds of accrual into a clinical trial compared to patients aged 40–49 years (OR: 0.25; CI: 0.118–0.552, *p* < 0.001) In addition, compared to White patients, Hispanic patients with prostate cancer also had lower odds of accrual into a clinical trial. (OR: 0.35; CI: 0.161–0.744, *p*=0.007) Relative to White patients, similar disparities were observed in lower odd ratios for patients who had race designated as other (OR: 0.524; CI: 0.295–0.931, *p*=0.028) and for those with missing racial designation (OR: 0.253; CI 0.137–0.466, *p* < 0.001).

## 4. Discussion

Clinical trials are essential to advance care in modern medicine. As such, addressing racial disparities in clinical trial enrollment has been an ongoing and well-recognized healthcare policy concern. We focus on this need for increased racial diversity within clinical trials specifically for patients with advanced prostate cancer given the racial disparities that persist for outcomes. To develop effective reporting and interventions to promote diversity and heterogeneity in clinical trial enrollment for highly prevalent malignancies, it is essential to have accurate estimates of clinical trial accrual across different races and disease severity to ensure that results have generalizable clinical implications across all patient populations [7]. Against this backdrop, our study provides several key findings that will inform health policy for equitable access and accrual for clinical trials in advanced or metastatic prostate cancer.

First, our study clearly demonstrates that Hispanic/Latino patients with advanced prostate cancer continue to face significant challenges and barriers to clinical trial enrollment in a population-based cohort of oncology practices across the United States. These results have important implications since our study captures racial disparities in clinical trial enrollment affecting only Hispanic/Latino patients with advanced prostate cancer from the same at-risk patient population. Prior studies have shown that African American and Hispanic/Latino men have markedly lower rates of clinical trial enrollment for prostate cancer [4, 8, 12, 13]. However, it is essential to acknowledge the differences in methodology for our study compared to the other population-based studies. More specifically, one possible concern is how prior studies used regions to establish the population estimates. However, this may in fact differ from the patients truly eligible for enrollment into clinical trials for several plausible reasons [8]. For example, geographic variations have been shown to correlate with racial disparities, in particular for African American men regarding access to care and stage at diagnosis and overall clinical severity at the time of diagnosis [2, 10, 14]. Other studies have queried publicly reported therapeutic trials to critically examine the number of trials reporting race and summed the distribution of patients by race [3, 13, 15, 16]. A limitation of such studies is understanding the barriers to clinical trials, such as access and mistrust [17]. It is also possible that prior studies may have different inferences due to these methodologic concerns about defining the eligible patient population at risk with aggressive prostate cancer. Finally, other studies may have missed current trends as we have analyzed the data through 2023.

Our study addresses this possible methodologic concern by ensuring that patients who were enrolled into clinical trials for advanced prostate cancer came from the same at-risk patient population and includes representation from the community oncology practices throughout the United States. Prior studies have underscored the lack of diversity in prostate cancer clinical trial enrollment for African American men [4]. Studies have also demonstrated disparities among Hispanic/Latino patients for therapeutic drug and nonspecific surgical clinical trials [4, 18] From our cohort, we were able to demonstrate the lack of involvement of Hispanic/Latino men in clinical trials specifically for patients with advanced prostate cancer. Several more recent studies have produced similar results. For instance, Javier-DesLoges et al. used the National Cancer Institute's Clinical Data Update System from 2000 to 2017 and found that Hispanic and African American with prostate cancer had lower odds of enrollment compared to White patients after adjusting for the cancer incidence for each race over time (both *p* < 0.001) [4]. Our data demonstrate that this trend has persisted beyond 2017 through 2023 despite the increased awareness of this phenomenon within clinical trial accrual.

Against this backdrop, understanding the reasoning that contributes and explains the barriers to clinical trial enrollment of men diagnosed with prostate cancer who identify as Hispanic/Latino or other is essential to develop effective interventions to address this disparity. One concern, however, is the limited evidence providing a comprehensive conceptual framework in understanding the reasoning behind these disparities and developing effective interventions to address them. In a prospective study of patients who were approached to enroll in oncologic clinical trials, Lara et al. found that patients who declined to enroll often cited a desire for other treatments or transportation to tertiary hospitals [19]. Other key factors that have been cited as likely barriers to equitable enrollment for cancer clinical trials also include access, mistrust, and financial toxicity [17, 20]. For all clinical trials, Leiter et al. used the NCI Health Information National Trends Survey, a nationally representative sample of adults in the U.S., and reported that lower educational levels and Hispanic/Latino ethnicity were associated with lower awareness of clinical trial availability [21]. There is also the possibility that there could be a language barrier that may be hindering accrual in this population as well. Taken together, defining the key components of a conceptual framework behind the racial disparities for clinical trial enrollment would allow for more ideal targeting to address racial disparities in clinical trials and ultimately lead to greater generalizability of diagnostic or therapeutic trials.

Second, another salient finding from our contemporary study with results reported up to 2023 showed that participation of adults in clinical trials remained low. Decreased participation overall and across minority patients has been initially shown to be less than 2% in 2004 [5]. There is little evidence to demonstrate that patient participation in clinical trials has substantively changed over time. Indeed, it is estimated that only 1%–4% of adult cancer patients participate in clinical trials [22]. Furthermore, patients who are enrolled in trials may have limited generalizability due to the lack of older patients and women, both of whom have been associated with lower clinical trial participation [4, 16, 23]. Moving forward, it will be essential to increase attention on increasing clinical trial participation for patients of all ages and addressing the modifiable barriers within these studies [7, 22].

It is also necessary to highlight the limitations of our study. While our study describes clinical trial enrollment from a contemporary population-based cohort of prostate cancer patients, CLQ-DB does not provide details about the specific type of clinical trial, diagnostic, therapeutic, and surgical or pharmaceutical, for patients who were accrued. CLQ-DB also does not provide details on the phase of the trials (Phase I vs. II vs. III trials). It also does not include data from where the patient received their care (academic vs. community practice). Another limitation of our study includes the limited sample size of patients that were encompassed in the CLQ-DB. Using data from the ASCO, approximately 858,000 men were diagnosed with advanced prostate cancer from 2011 to 2023. This means we were able to include and study roughly 2% of these patients within the CLQ-DB. Also, our study design may have been limited due to the lack of clinical granularity about the severity of prostate cancer metastasis. In addition, limited information about the inclusion criteria for clinical trials may also have overestimated the lack of participation in our study. However, it is also essential to recognize that we provide a novel study design where patients enrolled into clinical trials come from the same study population of advanced prostate cancer.

In summary, our results provide critical information about the current rates of clinical trial participation and its potential barriers to enrollment from a contemporary cohort from 2011 to 2023. Over the last decade, clinical trial participation has remained low with little evidence to suggest that it is increasing over time. Moreover, Hispanic/Latino patients, but not Black patients, with advanced prostate cancer from a large national database of oncology practices across the United States have low levels of clinical trial participation. Increased attention to developing and implementing effective incentives and interventions is needed to address modifiable barriers for trial enrollment specific to minority populations. Another urgent need is to develop reliable and validated mechanisms to accurately capture the participation of adult cancer patients in clinical trials across races and other social determinants of health. It is crucial to ensure that results from clinical trials are generalizable and applicable to a diverse prostate cancer patient population.

## Figures and Tables

**Table 1:** Patient and clinical characteristics of patients enrolled into a clinical trial.

Study characteristics	No clinical trial enrollment (*n* = 16,578; 97.3%) *n* (%)	Clinical trial enrollment (*n* = 450; 2.7%) *n* (%)	*p* value	Overall (*n* = 17,028) *n* (%)
Patient age (years)				
40–49	206 (1.2)	9 (2)	< 0.0001	215 (1.3)
50–59	2120 (12.8)	73 (16.2)		2193 (12.9)
60–69	5659 (34.1)	183 (40.7)		5842 (34.3)
70–79	5600 (33.8)	151 (33.6)		5751 (33.8)
80–90	2993 (18.1)	34 (7.6)		3027 (17.8)
Race				
Asian	353 (2.1)	25 (5.6)	< 0.0001	378 (2.2)
Black	2727 (16.4)	64 (14.2)		2791 (16.4)
Hispanic	454 (2.7)	7 (1.6)		461 (2.7)
White	11,195 (67.5)	330 (73.3)		11,525 (67.7)
Other	718 (4.3)	13 (2.9)		731 (4.3)
Missing	1131 (6.8)	11 (2.4)		1142 (6.7)
Region				
Midwest	4180 (25.2)	35 (7.8)	< 0.0001	4215 (24.8)
Northeast	2027 (12.2)	22 (4.9)		2049 (12)
South	6573 (39.6)	124 (27.6)		6697 (39.3)
West	3462 (20.9)	263 (58.4)		3725 (21.9)
Missing	336 (2)	6 (1.3)		342 (2)
Year of diagnosis				
2011	667 (4)	11 (2.4)	< 0.0001	678 (4)
2012	874 (5.3)	6 (1.3)		880 (5.2)
2013	932 (5.6)	24 (5.3)		956 (5.6)
2014	1178 (7.1)	28 (6.2)		1206 (7.1)
2015	1356 (8.2)	34 (7.6)		1390 (8.2)
2016	1638 (9.9)	35 (7.8)		1673 (9.8)
2017	1808 (10.9)	68 (15.1)		1876 (11)
2018	1927 (11.6)	104 (23.1)		2031 (11.9)
2019	1775 (10.7)	48 (10.7)		1823 (10.7)
2020	1346 (8.1)	35 (7.8)		1381 (8.1)
2021	1219 (7.4)	27 (6)		1246 (7.3)
2022	1281 (7.7)	18 (4)		1299 (7.6)
2023	577 (3.5)	12 (2.7)		589 (3.5)
Gleason score				
7	439 (2.6)	2 (0.4)	0.0008	441 (2.6)
8–10	1468 (8.9)	26 (5.8)		1494 (8.8)
Missing	14,671 (88.5)	422 (93.8)		15,093 (88.6)
PSA ≥ 10	7714 (46.5)	171 (38)	0.0003	7885 (46.3)
Clinical stage				
N0M1	2643 (15.9)	61 (13.6)	0.002	2704 (15.9)
N1 + M0	3641 (22)	126 (28)		3767 (22.1)
N1 + M1	3613 (21.8)	113 (25.1)		3726 (21.9)
N1 + MX	577 (3.5)	16 (3.6)		593 (3.5)
NXM1	6104 (36.8)	134 (29.8)		6238 (36.6)

**Table 2:** Multivariable logistic regression of enrollment into a clinical trial (*n* = 17,028).

Covariate (referent)	OR (95% confidence interval)	*p* value
Patient age (40–49 years)		
50–59	0.78 (0.38, 1.62)	0.51
60–69	0.70 (0.35, 1.42)	0.32
70–79	0.56 (0.28, 1.14)	0.11
80–90	0.26 (0.12, 0.55)	< 0.01
Race (White)		
Asian	1.20 (0.77, 1.86)	0.42
Black	1.03 (0.77, 1.38)	0.83
Hispanic	0.35 (0.16, 0.74)	0.01
Other	0.52 (0.30, 0.93)	0.03
Missing	0.25 (0.14, 0.47)	< 0.001
Region (northeast)		
Midwest	0.54 (0.31, 0.93)	0.03
South	1.21 (0.75, 1.93)	0.44
West	4.62 (2.94, 7.26)	< 0.01
Missing	1.53 (0.61, 3.85)	0.37
Year of diagnosis (2011)		
2012	0.45 (0.17, 1.24)	0.12
2013	1.71 (0.82, 3.55)	0.15
2014	1.52 (0.74, 3.09)	0.25
2015	1.80 (0.90, 3.61)	0.10
2016	1.47 (0.73, 2.93)	0.28
2017	2.61 (1.36, 5.02)	< 0.01
2018	3.47 (1.83, 6.57)	< 0.01
2019	2.02 (1.03, 3.95)	0.04
2020	1.86 (0.93, 3.74)	0.08
2021	1.86 (0.90, 3.82)	0.09
2022	1.28 (0.60, 2.77)	0.53
2023	1.96 (0.84, 4.55)	0.12
Gleason score (7)		
8–10	0.26 (0.06, 1.09)	0.07
Missing	1.29 (0.85, 1.96)	0.23
PSA ≥ 10	0.75 (0.61, 0.91)	0.01
Clinical stage (N1 and M0)		
N0M1	0.79 (0.58, 1.09)	0.15
N1 and M1	1.133 (0.87, 1.49)	0.37
N1 and MX	0.90 (0.52, 1.55)	0.70
NXM1	0.79 (0.61, 1.02)	0.07

## Data Availability

The data that support the findings of this study are available from CancerLinQ upon reasonable request.
